# On the Formation of Tumours

**Published:** 1831-01-01

**Authors:** 


A Short Tract on the Formation of Tumours, and the Peculiarities that are met with in the Structure of those that have become Cancerous; with their Mode of Treatment. By *Sir Everard Home*, Bart., &c. &c. 8vo. pp. 98, with several Plates. Sep. 1830.

The venerable and talented author of this little volume observes that, as this is the last of his professional labours that will be submitted to the public, he cannot, at the age of 74, make a better preface than by "an humble prayer of grateful acknowledgement to the all-wise Creator, who has permitted him to continue the investigation of his most wonderful works for so long a period, and thus enabled him, in many instances, to alleviate the miseries of suffering humanity, and to glorify the name of the Author of our existence." This is at least a pious preface, and we hope the writer of it will yet enjoy many years of the otium cum dignitate, now that his philosophical and professional labours have honourably closed.

In 1800, and in 1805, Sir E. published in the Transactions of a Society, &c. an account of tumours found in the thecæ of nerves, and a tract on cancer. "In youth, he says, we only publish our professional acquirements—in age we can lay before the public the result of our own professional experience; and unless the *first* have appeared, the second cannot be justly appreciated." We do not quite understand the foregoing proposition. We imagine that the value of a man's professional experience in age might be appreciated, and justly appreciated, if he had never appeared before the public in youth.

Sir Everard conceives that all tumours are formed by depositions from the blood—and, in general, resemble in structure, more or less, the substance of the natural parts by which they are surrounded. Fatty tumours, he avers, are nothing more than deposits of fat in parts that have been slightly injured.

"Where the injury has been more severe, the materials of which the consequent tumours are composed differ according to the quantities and new combinations of the extravasated materials; but although unlike one another, still, however, in general, in their texture, they bear a resemblance to the healthy parts by which they are surrounded.

It is not only necessary for the surgeon to know the nature of the structure of the tumour, but in many cases it is important to examine with accuracy the parts surrounding it, and the mode in which it is connected with them.

There is a particular species of tumour, met with in the neck, and probably in other situations, which is contained in a cyst, and only loosely connected with it by small blood-vessels from its surface and cellular membrane. It is of a yellowish-white colour, and somewhat resembles a kidney. It is of a uniform texture; and when allowed to increase to a considerable size, some portions become more compact than others.

This tumour is very common; and in its removal it is only necessary to lay open the cyst, and disengage the tumour with the finger. This I have frequently done: but there is a tumour apparently similar, incased in a nerve, which requires a different mode of extirpation. I shall mention the cases of this kind which have come under my observation, and describe the mode of operation which should be employed for their removal." 5.

Sir E. relates the case of a tumour the size and shape of a pullet's egg, situated on the outer side of the biceps muscle of the right arm. It was moveable in all directions, and, on laying it bare, it was found to be inclosed in a nerve. The whole was removed; but the patient had no use afterwards of the thumb and forefinger, the skin of which became dry, rough, and scaly. The tumour covered by nerve, appeared to consist of serpentine fibres, externally, with a homogeneous substance within. Another case somewhat similar is stated. These tumours have this peculiarity—when moved laterally no pain is felt—but if moved in any other direction, acute pain is complained of.

Sir E. next proceeds to the narration of a very remarkable case that occurred in St. George's Hospital, 14 years ago, where the tumour arose from the diploe of the cranium, in consequence of external injury, and in its increase made its way through the external table, without injuring the internal. The tumour was twice the size of the poor woman's head, and the surgeons thought an operation highly dangerous.

"After maturely weighing all the circumstances, she cheerfully submitted to the operation, which I performed on the 9th of October, 1816, in the theatre of St. George's Hospital. The operation was begun by a crucial incision through the integuments down to the surface of the tumour; the four flaps of skin were turned back; and all the soft parts of the tumour, which consisted of fat mixed with a steatomatous substance, were removed; and as doing this had occupied a considerable time, the skin was brought over the remaining tumour, and the patient put to bed. The pain had not been severe, and was submitted to with great fortitude. On the next day the skin was turned back; the bony rim surrounding the base of the remaining tumour, formed by the external table, was exposed all round; and, as it was close to the orbit, a saw was so contrived that its blade could be passed between them, having an iron bow fixed upon the opposite side of the blade instead of behind; so that there was no impediment to the working of the saw till the extensive base of the tumour, that consisted wholly of bone, was sawed through. The integuments were then brought forwards; and, although they were at first so much too large as to be thrown into folds, they very soon contracted, and in a few days did not extend beyond the surface on which they were laid; and the parts healed in the same manner as any other wound, leaving a firm cicatrix, with a more regular surface than there was reason to expect. In the course of the healing of the parts, no symptoms, either local or constitutional, were produced." 15.

The female was detained as a servant at Chelsea, in order that the result of the operation might be noted. She was afterwards admitted as a nurse in St. George's Hospital, where she remains in good health.

The next subject discussed is cancer. This disease, says Sir E. has hitherto been considered as one originating from a poison generated in different parts of the body, from accidental or other causes.

"As the same parts in different individuals, under similar circumstances of violence, do not always form cancerous tumours, when they do so, it must arise from a peculiarity of constitution disposing the injured parts to take on this disease; and therefore the tumour, in its origin, cannot be cancerous." 18.

Sir E. rebuts the idea that any disease can be hereditary—but comes to nearly the same thing by admitting a *predisposition* to be transmitted from parent to progeny. When the muscular and tendinous fibres of the gastrocnemius muscle are lacerated, the ruptured vessels bleed and form a tumour, which is sometimes absorbed—sometimes becomes cancerous. The late John Hunter, in consequence of throwing a stone with over-exertion, strained his pectoral muscle—the lacerated fibres produced hæmorrhage and swelling—he became faint—and required rest for some time, but ultimately did well.

"A gentleman who had a similar accident, followed by a tumour in the part, which he did not believe of any consequence, insured his life, and declared himself labouring under no disease. He afterwards died from a cancer formed in that tumour. Mr. Cline was consulted, and said that the tumour in the pectoral muscle was a cancer, and had been so from its first formation. In this opinion he was supported by another surgeon; and therefore the office refused paying the insurance, as the gentleman died in consequence of the fungous excrescence which this tumour afterwards produced. The case came before Sir William Grant, then Master of the Rolls, who was staggered by Mr. Cline's affidavit, but not convinced, and called upon me to know my opinion. I stated, that if Mr. Cline was correct, all such accidents would be immediately followed by cancer, which I knew was not the case, and gave him several instances in confirmation of my assertion, in particular, that of Mr. Hunter; and the gentleman's executors gained their cause: since Mr. Cline could bring no proof when the cancerous disposition first took place; and as the gentleman, at the time he took the oath, could have no knowledge that the swelling brought on by the accident could be the forerunner of any disease." 21.

Sir E. next indulges in various theoretical speculations respecting the origin and formation of tumours, on aneurisms, hydatids, and cancers, which we confess we are not much inclined to analyze. In the year 1773, Sir E. began his studies under John Hunter, and, as the treatment of scirrhus and cancer had baffled the attempts of the regular surgeons, it fell into the hands of empirics. Their practice consisted in the destruction of the morbid growths by different kinds of caustics, especially the arsenical. Like St. John Long's liniment, these poisons and escharotics were believed to act only on the diseased structures, and to spare all sound parts. "But experience proving the want of success of this practice in cases of confirmed cancer, and the cruel torments produced by such applications lasting for a great length of time, it fell into disuse." The knife was therefore preferred by regular surgeons, who hoped, when the whole of the tumour was removed, that the disease would not return. That the surgeon was often deceived in this expectation we need not now say. Mr. Hunter was very much employed in operations, and particularly in the dissecting out of tumours, from his paramount anatomical knowledge. These opportunities of observing were extended to Sir Everard, and continued in his own person for 57 years—no trifling experience. Sir E. has now selected from a great and overwhelming case-book, a series of cases exemplifying the more usual progress of cancer, both where it was allowed to run its course unmolested, and also where it was made the subject of surgical operation. These cases are related in such a brief manner that it is impossible to abbreviate them; yet, as authentic records of facts, we are loth to pass them over unnoticed. We shall therefore include a good leash of them in one extract.

"*Cases of Cancer of the Breast that came under my own immediate Observation.*

1. A lady, when forty-eight years of age, had a lump in the left breast, considerably advanced towards ulceration. The glands in the axilla and above the clavicle were swelled and indurated; the arm was swelled, with pain in the shoulder and back.

When twenty-eight years old she had a small tumour, the size of the end of the finger, which remained stationary for six years, at thirty-five years of age grew larger, and occasionally gave pain. It afterwards rapidly increased, and arrived at its present state of a confirmed cancer, which was considered beyond the reach of an operation; and it terminated in the death of the patient.

2. A lady, between fifty and sixty years of age, had a cancerous tumour in the breast, moveable upon the pectoral muscle. This was thought by the surgeon in the country a case for operation; but Mr. Cline, on examining it, was of a contrary opinion. In about two months from this period the arm had become much swollen, and at this time my opinion was requested, which accorded with that of Mr. Cline; but we were unable to convince the surgeon in the country that he was not in the right. He said that the swelling in the arm made no part of the disease, and was only a sympathetic affection, which could be readily reduced. I told him, that as soon as he succeeded in reducing the swelling of the arm, neither Mr. Cline nor myself would object to the operation upon the breast.

By means of bandages he was enabled to diminish the size of the arm, but not to prevent the return of the swelling to the same degree as before, when the bandages were omitted. The disease continued to increase, and in a few months the patient died.

3. A lady, fifty-eight years of age, had a tumour in the breast, which for nine or ten years had been growing to its present size. Several glands in the axilla were enlarged; the tumour itself made slow progress, but the skin, which firmly adhered to the tumour, and had an appearance of being tucked down upon it, had in the neighbourhood become studded over with small tumours, resembling split peas: they first appeared there, but in nine months they were met with all over the body, on the opposite side as well as that on which the tumour had formed. They were in no place close together, being about an inch apart, and nearly the same in size, but rather larger near the original disease. They gave a considerable degree of uneasiness, and her health was much impaired. In a few months she died. The tumour had previously become painful; frequent retchings had been produced, and her stomach retained little or no food.

It is a question whether the smaller tumours in the skin were cancerous, although consequences of that disease, since they are met with in cases in which no previous cancer had existed.

4. A lady of a delicate habit, who, before she was 37, had several children, and had had milk abscesses in both breasts, found a small tumour in her left breast; besides this, the glands in the axilla were enlarged. This tumour I did not believe cancerous, as the glands in the opposite axilla were also enlarged. The tumour increased in size in the course of a year; and in another year was still larger. In the third year the glands in the axilla were more swelled and painful, and a small tumour formed in the skin, near the original one. In the fourth year all the symptoms increased, and a second tumour formed in the skin. An inflammation in the lungs, to which she was subject, now came on, from this time she never noticed her breast, in which the symptoms remained stationary; and after an illness of four months she died.

An operation would have hurried on the disease, and caused her death at a much earlier period.

5. A lady, fifty-seven years of age, had a small tumour in the breast, without pain, the size of a pea. In two years it had scarcely enlarged. At that time she received a blow upon it, and it afterwards rapidly increased, with occasional pain. Six months after this I saw it. The tumour, which was situated above the nipple, was too large and too firmly connected with the surrounding parts to encourage an operation proving successful. I saw it occasionally: its increase was uniform; no glands in the axilla became enlarged, but one swelled above the clavicle. In six months she had a violent inflammation in her lungs. On her recovery the tumour had become very large. After another year a tumour formed between the breast and clavicle, and two small ones above it. At this time an empiric gave her promise of a cure; but she was to give him a guinea a day till it was effected. I begged to have an interview, and told him that if he failed I should ruin his character by my exposure of it; and as the glands above the clavicle were affected, he could not succeed: in this way the attempt was abandoned.

In two years more the original tumour became a sore: this was five years after it had been struck. The ulcerated surface was about the size of half a crown: it discharged a considerable quantity of limpid fluid: the ulceration increased rapidly, the edges rising higher than the surface of the sore. In two months a bleeding took place, which was readily suppressed: this returned periodically every three weeks, to the quantity of eight ounces.

At the end of the sixth year the ulcer became very large; and from the thickness of its margin, covered with an imperfectly formed cuticle, it acquired a great depth. Her general health became impaired; and the pain was so severe that she took two hundred drops of laudanum twice in the day, to afford her intervals of ease.

At the end of the ninth year she died, worn out by the repeated bleedings and the progress of the disease. The only treatment made use of was powdered extract of hemlock, which for years considerably diminished the pain; so much so, that when some which had been prepared with great attention was expended, and that made in the ordinary way was substituted, she complained that it had lost its beneficial effect. Had she employed the empiric, her life would have been shortened, and her income, which was small, eaten up.

6. A lady, who had occasionally matter, blood, or bloody water issue from the nipple, had, some months after, a tumour formed, and the nipple ulcerated; the glands in the axilla swelled, and all the symptoms of cancer came on, of which she died.

7. A woman was received into St. George's Hospital with a small tumour at the basis of the nipple, which was very moveable. The first symptom of the disease was blood oozing from the ducts in the nipple. It was removed with the surrounding parts. The tumour, when examined after removal, was found to be entirely circumscribed, and appeared to be no part of the gland of the breast, but a newly-formed structure; and I never heard of the disease having returned.

8. A lady, twenty-three years of age, had a tumour in the breast, hard to the feel, giving occasional pain: it had continued for a year, when Mr. Hunter extirpated it with the parts surrounding it. Upon examination of its structure, it was found to be a solid tumour, distinct from the neighbouring parts, to which it was only slightly attached.

It is impossible to tell whether it would have ultimately become cancerous; but there is every reason to believe that, as she advanced in life, it would have acquired a cancerous disposition.

9. A lady, thirty-five years old, had two small tumours in the left breast, at six inches distance, on the upper part, without pain or uneasiness. I stated that, in my opinion, they were not malignant. In two years they had not enlarged, but were at times painful. The patient wished them removed, and the operation was performed. When examined, they were found to be circumscribed; and when the covering was divided the substance expanded itself.

10. A woman, fifty years of age, who had a tumour in the breast, which had not arrived at a large size, and was unaccompanied with enlargement of the glands in the axilla; it was therefore extirpated by Mr. Hunter, in 1774. The wound was slow in healing, and a knot the size of a pin's head appeared on the cut edge of the skin; in two days a second knot appeared, at the distance of three fourths of an inch: these increased rapidly in size, and were destroyed by caustic. The wound healed, and she never afterwards had return of the disease, but died of a dropsy, seven years after the operation.

This case proves that, in the first instance, the appearance of small excrescences on the cicatrix of the wound, made in the removal of cancerous tumours, is not always of a morbid nature, and capable of carrying on the disease.

11. A lady, thirty-two years of age, the mother of several children, discovered by accident a tumour in the breast. As the tumour was movable, means were taken to disperse it; but these proving ineffectual, I was consulted, and advised its removal, which was acceded to. At the time of the operation the tumour moved freely in one direction, but was more confined in that of the fibres to which it was found to be attached, and part of that muscle removed along with it. The wound healed in three weeks. In six months after the operation, a fulness was felt in the pectoral muscle, attended with pain; in a twelvemonth a tumour had formed, and the skin was put on the stretch. The pain had become intolerable; the tumour daily increased; and, upon her being seized with vomiting, the lower part became discoloured from the rupture of the smaller vessels. A fortnight afterwards the skin broke, and a fungous excrescence appeared, covered with blood, from the vessels in the surface giving way. In about three weeks she died.

From this case we learn the difference which the disease of cancer puts on in a glandular structure and in a muscular one: it also explains to me the nature of the true hæmatoides, which is the effect of cancer when a muscle is the seat of the complaint." 49.

More than a dozen more cases are related by our author, but we do not deem it necessary to notice them.

After the mamma, Sir E. considers the tongue as most liable to cancerous affection. Any local complaint in this organ, he observes, is liable to take on the carcinomatous character—in other words, to propagate itself by affecting the contiguous glandular structure, "after which the disease is carried on by absorption." All such cases have a fatal termination, if the diseased parts are not early extirpated. When the operation is early performed, a cure has always been effected. Cases in illustration, chiefly from the practice of St. George's Hospital, are given by the author.

"Cancer in the testicle has two modes of making progress; the one by becoming a scirrhus, exceedingly hard, and contaminating the cord, and then the lymphatic glands, in its progress, as high as the loins. This is called a genuine cancer, and has been described accurately by several writers on surgery. There is, however, another disease, which, as it contaminates the neighbouring parts, and continues its course by means of the absorbent glands, may be considered not improperly as the same disease.

In either of these local diseases, the parts are not capable of contamination of the neighbouring parts till after those have long continued to have lost their healthy state; and when the organ is extirpated early, the patients, I believe, always get well. But this is a rare occurrence when the disease has completely established itself; and when it has, an operation, or any other violence committed upon the parts, by its irritation, accelerates the progress of the disease.

A gentleman had an occasional swelling in one of his testicles, which subsided, but returned again, and in the end became permanent, extending some way along the cord. When it was in this state, it was extirpated; the skin that adhered to the testicle was freely removed, and the cord was included in the ligature, as high as the abdominal ring:—all went on well. About nine months after the operation, a swelling under the upper part of the cicatrix was observed, which appeared, both to Mr. Pott and Mr. Hunter, to be a lymphathic gland. This increased in size, and with others that appeared formed one mass: a degree of fluctuation was felt, ulceration took place, and a fungus shot out, which often bled, and he died.

In this case the return was not in the line of absorption nor cord; and the cicatrix never gave way. To account for this return, the glands in the groin must have been affected by the diseased skin of the scrotum.

This is an uncommon circumstance, and surgeons have not hitherto taken the risk of the disease being carried on from the scrotum into the account, when an operation is the question under consideration." 73.

Cancer in other parts besides glands is frequently met with. In some cases it is the immediate consequence of accident—in others, it is the effect of the parts having long-continued in a diseased state, till a certain advance of years render them more susceptible of the carcinomatous action.

"We have from this a confirmation of no cancerous disease ever being so in its origin; and learn, that, when parts have been long in a diseased state, we have no security against their not ultimately taking on a cancerous action: but in all such cases, there must be a peculiarity of constitution existing, previously to the parts undergoing such a change." 77.

We confess we cannot so clearly see the proof in these cases, that no disease is ever cancerous from the beginning. When Sir E. tells us that in all these instances, there must be "a peculiarity of constitution existing," what is this but another term for cancerous leaven in the constitution? It is only rendering *obscurum obscurius*. We shall conclude this article with the following extract which also closes the volume.

"Having stated the principal cases of this disease that have come under my observation, so as to explain its nature and symptoms, and their progress in particular instances; and having had the opportunities continued to me, for half a century, of paying attention to this subject, I am sorry to add, that very little progress has been made, either towards a cure or prevention of the disease taking place. Many tumours like those that formerly were, by violent applications, rendered true cancers, now never take on the disease; but no one is enabled, from such evidence, to prove that their more ordinary course would ever have led to such a termination. The progress of the symptoms having been decidedly retarded, and rendered less violent, is sufficient evidence that such mild means have been employed with great advantage, in cases which were of a dubious nature.

The treatment in my own practice, that calls forth this commendation, is the internal and external use of hemlock; and, in proof of its efficacy, in some cases when the medicine was left off, the symptoms became more violent; and when resumed, abated. Also, when the powder of the leaves was prepared at the proper season, and the light entirely excluded while the drying of it was carried on, even in confirmed cancerous ulcers, benefit was derived in so great a degree, that the patient could ascertain from increase of pain some change had taken place in the application, when powder less accurately prepared was used as a substitute for the other. I have even prevented the operation when the day had been fixed; and the patient lived for months under this palliative mode of treatment, without any progress of the tumour, and was carried off by epileptic fits. In many cases the swelling diminished, and in others remained stationary for years, and never afterwards made any advance: so that I am convinced that I had before been too much, alarmed, and frequently came to an operation before it was required.

It is now fifteen years since I was taught by experience that the sarsaparilla, in the form of decoction, has not the same powers of a restorative medicine as in the form of a powder, to which heat had not been applied. Mr. Cline had ascertained the same fact: this I found when in consultation upon the case of a young gentleman, who had his uvula destroyed by ulceration. In this case several medical men gave an opinion in favour of nitric acid, and of the use of the decoction of sarsa. Mr. Cline and myself warmly expressed ourselves in favour of the sarsa in powder; and our suggestions were adopted. In the course of four days the ulceration had been restrained by its use; and in less than a week the patient was allowed to be out of danger. From that time I never employed the sarsaparilla that had been exposed to heat, either by the fire, or the sun's rays in the window of an apothecary's shop.

In a case of ulceration of the palate and uvula, for which the patient had taken nineteen quarts of strong decoction of sarsaparilla, under the direction of one of the most repectable surgeons in Edinburgh, who made up, in his own house, the medicines he employed,—and therefore there could be no doubt of their being of the best kind, and prepared with every proper care,—the ulceration was still going on, and he came to London with the twentieth quart bottle in the carriage. Upon taking two drachms of the powdered root, freshly ground, three times a day, he recovered entirely in less than fourteen days." 97.

We now take leave of Sir Everard Home, and regret to learn from his own mouth, that it is a *final adieu!* There is something in that word which renders parting from an enemy somewhat painful—and still more when we are shaking hands for the last time with one whom we respect or venerate! Such separations, however, are the daily lot of mortality in this sublunary scene—and the nearer man approaches the awful goal, the more numerous they become!

